# When little can do more: the case for investing in mental healthcare in Ghana

**DOI:** 10.11604/pamj.2023.45.86.40409

**Published:** 2023-06-16

**Authors:** Joel Agorinya, Cephas Avoka, Luchuo Engelbert Bain

**Affiliations:** 1 Accra Psychiatric Hospital, Accra, Ghana,; 2 Faculty of Public Health, Ghana College of Physicians and Surgeons, Accra, Ghana,; 3 Department of Psychology, Faculty of Humanities, University of Johannesburg, Auckland Park, South Africa,; 4 International Development Research Centre (IDRC), Ottawa, Canada

**Keywords:** Ghana, investment, mental healthcare

## To the editors of the Pan African Medical Journal

About 21% of adults in Ghana suffer from moderate-severe psychological distress, leading to unemployment and productivity losses to 7% of Ghana´s gross domestic product (GDP) [1]. In two recently published reports on mental healthcare in Ghana, the World Health Organization- Assessment Instrument for Mental Health Systems (WHO-AIMS) 2020 and the World Health Organization Special Initiative for Mental Health Situational Assessment 2021 [2,3], key mental health considerations of public health and policy relevance draw our attention. Despite the significant strides Ghana has achieved over the years, the goal of creating an effective and comprehensive mental health service delivery infrastructure and workforce is far from complete. For instance, the current mental health service delivery architecture is skewed, with the Southern part advantaged. All three psychiatric hospitals in Ghana are in the South, with only three out of about sixty psychiatrists working in small psychiatric departments in the North. As a result, some indigenes have to travel over 700 km by road to seek care. This calls for attention to the equality and equity dimensions of health planning. The three psychiatric hospitals in Ghana have a combined capacity of 3.8 beds per 100,000 [3]. Additionally, whilst some low- and middle-income countries (LMICs) are investing about 4.0% of health expenditure in mental health, Ghana is investing 3% [4]. Another major challenge is low treatment coverage for mood disorders, with only 0.61% of persons with major depressive disorder receiving treatment. This rate is lower than the average of 16.8%-21.4% for some low-middle-income countries [5]. Furthermore, there are numerous challenges with the availability of psychotropic medications. This is a massive setback because Ghana has an overreliance on medical treatments due to a shortage of human resources to provide psychosocial services. In addition, most medications are paid out-of-pocket because Ghana´s national health insurance scheme does not cover them. Additionally, only 7% of health research in Ghana is specific to mental health. Furthermore, the data submitted to the government from health facilities are of low quality [2] and thus unreliable for research purposes, posing significant challenges to the quality and generalizability of the research output.

Despite the limited investment, Ghana has made significant strides in mental health. The Ministry of Health increased its expenditure on mental health from 1.4% to 3% over the past decade. As a result, the psychiatrist-to-patient ratio has improved from 0.07 to 0.13 per 100,000, and the mental health workforce has increased from 7.82 to 9.5 per 100,000 [2,6]. While these ratios remain inadequate, they serve as a model for other low- and middle-income countries. The enactment of the Mental Health Act established the Mental Health Authority and facilitated policy reviews. Ghana´s recent mental health policy emphasizes community care and effective governance structures for promoting mental well-being [7]. Efforts to deinstitutionalize care and shift towards community-based care have occurred in tandem with integrating mental healthcare into primary healthcare in Ghana. This has increased service delivery, with 423 outpatient mental health facilities dotted nationwide [2]. Another significant achievement from the limited investment in mental health service delivery is the collaboration with religious and faith-based healers and the steps taken to regulate their practices. The Ghana Health Service has an exemplary decentralized and functional healthcare system. Therefore, task shifting and sharing models can be integrated into this primary healthcare delivery model. More than 80% of persons with mental illness live in lower-middle-income countries [8], so more investment is needed in this sector. For example, in Ghana, 7% of our GDP is lost to psychological distress [1], even though the return on investment in mental healthcare ranges from Ghana cedis (GHC) GHS 2 to GHS 7 for each GHS 1 invested [9]. Meanwhile, the country´s twelve-year mental health policy estimates that the country needs between GHS 540 to GHS 720 million to meet the minimum package for mental healthcare [10]. Therefore, investments in the mental health sector should aim to build research capacity, scale up mental health professionals´ training, improve mental health infrastructure, and increase access to psychotropic medications. We acknowledge that others might present a grim view of Ghana´s mental healthcare system, especially compared to high-income countries. However, we prefer to remain optimistic and acknowledge the progress made, regardless of how little or slow it might be.

## Conclusion

The assessment of Ghana´s mental health landscape reveals the urgent need for increased investment to maintain the progress achieved in mental healthcare. However, the successful approaches implemented in Ghana can be valuable lessons for other low- and middle-income countries. In order to enhance mental health services, there is a requirement for investment in service delivery infrastructure, human resource development, financing, governance, health information systems, and research. These objectives align with Ghana´s mental health policy. This letter aims to raise awareness and generate more attention towards mental healthcare in Ghana, ultimately encouraging more significant investment and collaborations to improve service delivery outcomes.
